# Seeking Precise Protein-like Functions from Random Heteropolymer Ensemble and through Dimensionality Reduction

**DOI:** 10.1021/acscentsci.5c01382

**Published:** 2025-09-25

**Authors:** Guangqi Wu, Tianyi Jin, Haisen Zhou, Connor W. Coley, Alfredo Alexander-Katz, Hua Lu

**Affiliations:** † Beijing National Laboratory for Molecular Sciences, Center for Soft Matter Science and Engineering, Key Laboratory of Polymer Chemistry and Physics of Ministry of Education, College of Chemistry and Molecular Engineering, 12465 Peking University, Beijing 100871, China; ¶ Department of Chemical Engineering, 2167 Massachusetts Institute of Technology, Cambridge, Massachusetts 02139, United States; § Department of Materials Science and Engineering, 2167 Massachusetts Institute of Technology, Cambridge, Massachusetts 02139, United States; ∥ Department of Electrical Engineering and Computer Science, 2167 Massachusetts Institute of Technology, Cambridge, Massachusetts 02139, United States

## Abstract

Proteins achieve diverse biological functions through precise sequence-structure relationships, yet they can also function through statistical ensembles rather than as individual, static entities. Inspired by this paradigm, recent work has explored random heteropolymers (RHPs) as synthetic, scalable, and versatile protein mimetics. RHPs have been found to function as polymer ensembles capable of folding, binding, catalyzing, and stabilizing biomolecules with control over the monomer sequence. In this Outlook, we highlight recent advances in the discovery and mechanistic understanding of functional RHPs, emphasizing their emergent behaviors and utility across sustainability, human health, and pharmaceuticals. We discuss how autonomous experimentation, machine learning, and multiscale modeling are converging to accelerate design and discovery in this vast chemical space. By embracing statistical design principles, we propose a new framework for creating functional polymers that mirror biological systems.

## Graphical abstract

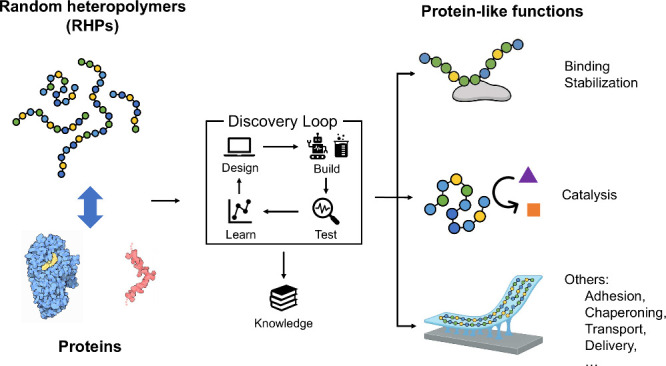

## 1 Introduction

Proteins, polypeptide chains composed of a defined set of amino acids, are the molecular workhorses of living organisms. From Anfinsen’s 1972 postulate that a protein’s amino acid sequence dictates its three-dimensional structure^1^ to recent breakthroughs in *in silico* protein structure predictors,^2–5^ the sequence-structure-function dogma of proteins has been investigated extensively. Proteins achieve functional diversity through evolution of their primary sequences.^6^ Such versatility, evolvability, and designability make proteins a foundational class of biomacromolecules in the pharmaceutical and biotechnology industries.

At the same time, nature offers compelling evidence that macromolecules lacking precise sequence or higher-order structure control can perform delicate protein-like functions, including but not limited to molecular recognition, catalysis, and phase separation. Many proteins do not operate as single, static, well-defined entities but as ensembles across multiple levels including the conformation level, sequence level, and molecular level (Figure 1). At the conformational level, one of the most prominent examples is the intrinsically disordered proteins or regions (IDPs/IDRs). Increasing evidence suggests that IDRs such as prion-like domains (PLDs), homorepeats or low complexity (LC) sequences, which lack stable secondary structures, govern diverse functions ranging from subcellular localization, macromolecular interactions, to disease progression as dynamic conformational ensembles.^8,9^ At the sequence level, certain proteins exhibit functions governed by their amino acid composition instead of their exact residue order. In other words, these proteins operate as sequence ensembles, and their evolution landscape appears to act at the compositional rather than individual sequence level. For instance, certain RNA-binding proteins (RBPs) retain their function after sequence shuffling, relying on global amino acid content rather than strict residue positioning.^10^ At the molecular level, protein function can arise from collections of molecules. For example, polyclonal antibodies, which consist of a diverse population of antibody molecules with different sequences, can collectively recognize and bind to various epitopes of the same antigen. Another example is biomolecular condensates formed by multiple proteins that interact through multivalent and often weak interactions, resulting in dynamic, phase-separated compartments without membrane boundaries that are essential for biological functions.^11,12^ These examples across multiple levels of molecular organization underscore the idea that functional precision can arise from diversity or randomness rather than uniformity or order. However, proteins and other biomacromolecules suffer from low stability under harsh or nonphysiological conditions, limited scalability for industrial manufacturing, and high costs. This prompts scientists to develop synthetic alternatives that mimic their functions.^13,14^

**Figure 1:**
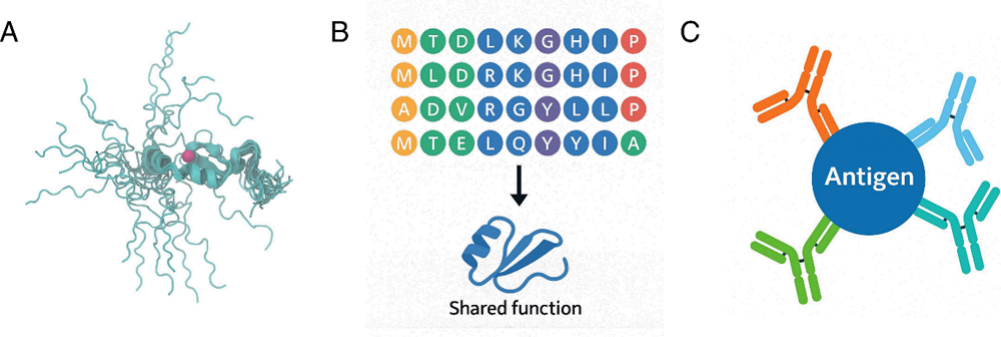
Illustration of functional protein ensembles at different levels: (A) Conformation level (PDB ID: 1ZR9, conformation ensemble of zinc finger protein with zinc ion in magenta. Conformation ensemble is rendered using VMD^7^ superimposed), (B) sequence level, and (C) molecular level.

Among these alternatives, synthetic polymers are particularly appealing for this purpose, as they not only exhibit a similar polymeric nature as proteins but can also offer key advantages over natural proteins, such as inherent chemical diversity, making them strong candidates for practical applications requiring protein-like functionality. The examples of protein ensembles raise an important question for protein-mimicking polymers: can polymers without precise sequence control be sufficient to achieve protein-like functions? If so, how do we design and develop such materials? Notable efforts on these materials have relied on specific protein-inspired structural design principles, such as single-chain nanoparticles (SCNPs) for enzyme mimetics^15–17^ and molecular imprinted polymers (MIPs) for antibody mimetics.^18,19^ More recently, random heteropolymers (RHPs), synthesized through the statistical copolymerization of three or more monomer types, have regained attention as promising candidates for mimicking and interacting with proteins. Since the 1970s, statistical copolymers have been used in commercial applications such as plastics [e.g., poly­(acrylonitrile-r-butadiene-r-styrene), ABS] and synthetic rubber [e.g., poly­(styrene-r-butadiene), SBR], and pharmaceuticals (e.g., glatiramer acetate)^20,21^ by the synergistic contributions from individual components. Due to stochastic polymerization used in their synthesis, RHPs operate as sequence ensembles, and compelling examples have demonstrated that functional behavior can emerge from composition alone. This has positioned them at a fascinating intersection between polymer science^22,23^ and protein science,^24–26^ making it an active area of research since the 1990s and one that will continue to attract significant interest in the future. These seminal theoretical and experimental studies were primarily based on binary monomer systems, whereas more recent advances have extended to heteropolymers with three or more monomers, thereby broadening the accessible sequence space and functional scope. This enormous chemical design space holds significant potential for discovering new functions and materials through variations in the monomer and composition. In this Outlook, we highlight recent advances in the development of RHPs as protein-mimicking materials, with a focus on emerging discovery strategies and their diverse potential applications.

## 2 Recent Development of Functional RHPs

### 2.1 Catalysis

Although RHPs may lack the well-defined active site architecture characteristic of natural enzymes (Fisher’s lock-and-key model), they can still create necessary local environments that facilitate interactions between substrates and catalytic centers through an induced-fit model.^29^ Polymer chains first collapse into globules through intra- or intermolecular noncovalent interactions or cross-linking. Catalytic functionalities can be introduced either through reactive monomers or the initiators themselves during the polymerization^30,31^ or via postpolymerization modification.^32^ The catalytic activities can be tuned by various parameters, such as the monomer compositions and the architecture of the polymers. RHPs have also demonstrated catalytic activity across a range of reactions. We recently reported that selenopolypeptide-based RHPs can mimic glutathione peroxidase activity by catalyzing the oxidation of glutathione through optimizing side chain structure and composition.^33^ The Knight group reported a polymer-based catalyst with triphenylphosphine-coordinated palladium for the Suzuki-Miyaura cross-coupling reaction.^34^ These studies collectively highlight the potential of RHPs to serve as tunable and robust platforms for developing synthetic catalytic systems beyond the constraints of natural enzyme architectures (Figure 2A).

**Figure 2:**
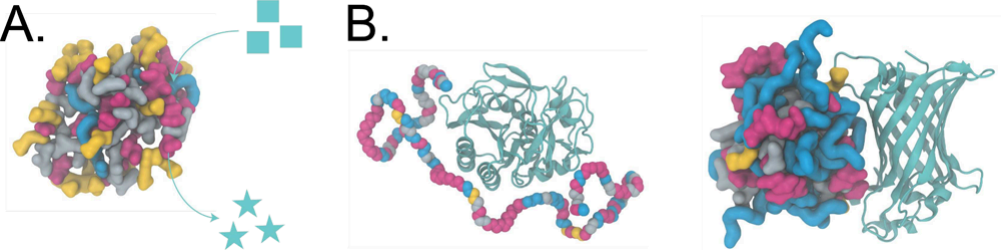
Schematic illustration of the functional versatility of RHPs, highlighting their ability to (A) catalyze small-molecule reactions and (B) bind to and stabilize enzymes in non-native environments. Specifically, RHPs can enhance the thermal stability of Proteinase K at elevated temperatures^27^ and preserve the structural integrity of the β-barrel membrane protein OmpLA in aqueous solution.^28^ The simulation snapshots are rendered using VMD.^7^ Protainase K (PDB ID: 1IC6) and bacterial OmpLA (PDB: 1QD5) are depicted in Cartoon representation, and polymers are in QuickSurf.

### 2.2 Protein Binding and Stabilization

RHPs can engage proteins through weak, noncovalent interactions, enabling selective targeting and functional modulation. Our recent work demonstrated that through composition optimization, RHPs can selectively bind to target protein tumor necrosis factor-alpha (TNFα) with single-digit nanomolar affinity, while showing minimal interaction with a control protein, human serum albumin (HSA).^35^ RHPs can also stabilize protein function under non-native and harsh conditions. The Xu group has demonstrated that by matching sequence segmental information between RHPs and proteins, RHPs can protect enzymes in organic solvents^36^ and under extreme processing conditions such as high temperature^28^ and melt extrusion.^27,37,38^ The Webb group, Gormley group, and we have shown that RHPs can enhance the thermal stability of enzymes through optimizing additional design parameters including chain lengths and blending ratios.^39,40,41^ Collectively, these findings underscore the potential of RHPs to serve as versatile, noncovalent protein binders and stabilizers across a range of challenging environments (Figure 2B).

### 2.3 Other Functions

RHPs have also been shown to mimic more sophisticated protein functions. The Xu group developed RHPs that act as synthetic single-molecule selective proton channels in liposomes,^42^ and mimic key aspects of biological fluidic systems such as creating condensate for DNA duplexation.^28^ In addition, RHPs have been demonstrated to disrupt protein-protein interactions, offering a means to modulate complex biological signaling pathways.^43^ The Gong group recently developed high-performance underwater adhesive hydrogels that mimic the function of natural adhesive proteins by integrating data mining from protein databases with experimentation and machine learning.^44^ These materials highlight the potential of RHP-inspired systems to achieve strong and selective binding through multivalent interactions, paralleling one of the core biochemical functions of proteins. In addition, stimuli-responsive behaviors, such as temperature sensitivity,^45,46^ have been introduced into RHP systems, enabling polymers to act as molecular switches. Meanwhile, introducing glycans into RHPs has emerged as a powerful strategy to mimic glycoproteins, providing a versatile platform for studying glycan-protein interactions and designing functional biomaterials for applications in therapeutic delivery, infection models, and vaccine development.^47^ These discoveries further expand the functional repertoire of RHPs and highlight their potential as versatile mimics of diverse protein behaviors.

## 3 Strategies for Discovering Functional RHPs

RHPs possess an exceptionally large and hierarchical design space, such as monomer structure, sequence pattern, composition, molecular weight, dispersity, and blending ratio (Figure 3A). Designing functional materials from this vast landscape requires efficient and scalable strategies. Scientists have been inspired by natural proteins to design the necessary components. As mentioned in the previous section, monomers responsible for solubility, folding, and catalytic reactivity can be carefully selected to generate enzyme mimetics. For protein binding and stabilization, surface characteristics such as three-dimensional geometries for target recognition in MIPs,^18,19^ or degree of chemical patching and sequence segmental information can be generalized to guide the selection of monomers and compositions.^28,36,48,49^ However, rational design becomes difficult when the key features responsible for the desired function are not understood or well-defined.

**Figure 3:**
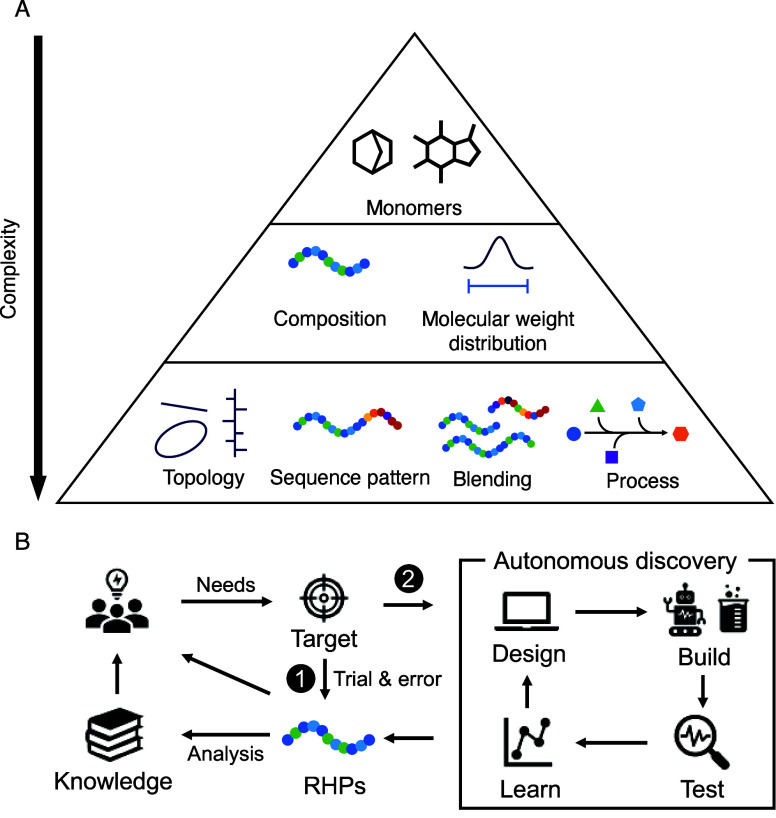
(A) Schematic illustration of the RHP design space, organized by increasing structural complexity, from monomer selection, overall composition, and molecular weight (MW) distribution, to higher-order features such as polymer topology, sequence pattern, compositional gradients, blending strategies, and process. This multiscale complexity underlies the rich functional diversity achievable in RHP materials. (B) Current discovery workflow for RHP materials. Material discovery can proceed through conventional approaches, which rely on human intuition and iterative trial-and-error experimentation, or through autonomous workflows that integrate automation and machine learning into design-build-test-learn cycles. Given the materials and associated data, retrospective analyses and experiments can be applied to uncover underlying structure-function relationships.

Compared to rational design, data-driven autonomous experimentation is particularly powerful to distill unexpected formulation-function relationships in complex systems where little prior knowledge exists

Recently, data-driven experiments have shown immense promise for the discovery of functional RHPs (Figure 3B). Conceptually, the discovery campaign of RHPs could be formulated as a black-box optimization problem: experiments are conducted iteratively, and the selection of conditions for each round is informed by data from previous iterations. Bayesian optimization (BO) has become a widely adopted strategy in this context due to its ability to balance exploration and exploitation while efficiently navigating high-dimensional design spaces. BO-guided autonomous platforms have successfully identified RHPs capable of stabilizing proteins,^40^ catalyzing chemical reactions,^33^ and serving as functional MRI agents.^50^ Alternatively, model-free approaches, such as genetic algorithms, offer powerful strategies for exploring high-dimensional design spaces, particularly when prior knowledge is limited. We recently developed a self-driving experimental platform coupled with a model-free genetic algorithm (GA).^41^ This approach enabled the discovery of RHP blends that outperformed all of the constituent polymers in functional assays. Compared with rational design, data-driven autonomous experimentation is particularly powerful to distill unexpected formulation-function relationships in complex systems where little prior knowledge exists. Importantly, we also highlight that with interpretable machine learning models, design rules can be extracted and generalized from what would otherwise be black-box optimization (vide infra).

## 4 Mechanistic Understanding of Functional RHPs

The discovery of novel polymers opens exciting possibilities but also raises fundamental questions: How do these polymer ensembles achieve their functions? What governs their behavior? Addressing these questions is essential for transforming phenomenological observations into a fundamental understanding. Unlike block copolymers,^51^ RHPs are inherently complex systems; each polymer chain differs from others in terms of sequence, composition, and molecular weight or degree of polymerization, making it difficult for traditional techniques such as cryo-EM to establish clear structure-function relationships at the molecular level. Techniques such as small-angle X-ray scattering (SAXS) and quartz crystal microbalance (QCM) analysis have been employed to study RHP-protein interactions by the Gormley Group,^52,53^ while hydrophobicity profiling of individual components using high-performance liquid chromatography (HPLC) has provided further insight.^54^ New characterization techniques are emerging to investigate this functional heterogeneity. For example, we have recently applied liquid-phase transmission electron microscopy (LP-EM) to directly visualize the real-time interactions between proteins and polymers.^35^ The Knight group recently developed a colorimetric assay capable of characterizing the conformational distribution of peptoid libraries.^55^ SRI International employed fiber-optic array scanning technology (FAST) to screen bead-based synthetic polymer libraries at a rate of about 5 million compounds per minute (approximately 83,000 Hz).^56^

Beyond analytical chemistry, subsampling strategies have been introduced to study the heterogeneity of these systems. For instance, once optimal monomer composition and molecular weight are identified in a fully random polymer, researchers can synthesize multiblock copolymers with the same composition but varied sequence distributions to probe functional contributions more precisely.^57^ The development of such high-throughput, structure-resolving methods represents a critical step toward the de novo design of disordered materials with complex emergent functions. While current experimental techniques can measure population-level properties, such as composition during the synthesis and purification process,^58^ acquiring information at the level of individual chains is still challenging.

Multiscale molecular simulations (MD) offer another complementary perspective; they have provided strong corroboration of experimental observations and generalizable design rules. They have also yielded important mechanistic insights into the structure, dynamics, and function of RHPs. We have developed an atomistic MD simulation protocol to study the self-assembly and dynamics of RHPs.^59^ This approach revealed key features of protein mimicry, including frozen backbone dynamics and mobile side groups,^59–62^ and dynamical heterogeneity.^62^ The glassy nature and associated internal friction significantly influence interfacial behavior.^28,63,64^ At the core is hydration frustration, where hydrophobic groups are hydrated, and polar/hydrophilic groups are dehydrated,^65^ mirroring the fundamentals of protein functions such as binding and catalysis.^66,67^ Notably, these protein-like properties are sequence-insensitive,^68^ suggesting that one-pot synthesis is sufficient to achieve functional convergence in RHPs.

Coarse-grained simulations by the Webb Group and Olvera de la Cruz Group have been applied to design and study sequence-structure relationships,^69,70^ multichain interactions for small-molecule uptake,^71,72^ and polymer-protein interactions^36,73,38^ allowing researchers to retain essential chemical features while balancing computational efficiency. As complexity increases, such as in polymer blend systems and when incorporating chemically specific properties like catalysis or metal binding, bottom-up molecular simulations may struggle to achieve both the necessary chemical accuracy and reasonable time scales.

Feature importance analysis offers a powerful means of identifying key design features from experimental data.^74^ Various methods have been used to interrogate high-dimensional RHP data sets, where human intuition alone may fall short in identifying critical variables. By highlighting critical features, these analyses help guide the design of follow-up experiments to retrospectively validate the potential underlying mechanisms. For example, SHapley Additive exPlanations (SHAP)^75^ has been employed by several groups to assess the relative importance of monomer identity and other molecular characteristics, such as monomer structures, composition, and degree of polymerization. The Gormley Group used SHAP and revealed that specific monomers and chain lengths were crucial for stabilizing enzyme activity.^40^ Similarly, the Reineke group applied SHAP analysis to uncover key polymer features that contribute to effective intracellular ribonucleoprotein delivery, providing valuable design principles for future polymer-based delivery systems.^76–79^ In addition to SHAP, dimensionality reduction and clustering techniques, such as principal component analysis (PCA),^28^ Uniform Manifold Approximation and Projection (UMAP),^50^ and t-distributed stochastic neighbor embedding (t-SNE),^33^ have also been applied to elucidate feature importance and visualize structural relationships within RHP data sets.

## 5 Outlook

As discussed above, recent works with RHPs point to the emergence of a new design paradigm for functional synthetic polymers: instead of emulating a single, precisely folded macromolecule, RHPs can be engineered as ensembles whose collective behavior yields protein-like functions. Continued progress in autonomous experimentation, data-driven modeling, and polymerization chemistry will accelerate the discovery of materials that will potentially address some of the crucial challenges facing human societies.

Instead of emulating a single, precisely folded macromolecule, RHPs can be engineered as ensembles whose collective behavior yields protein-like functions.

### 5.1 Application Outlook

Two general categories of RHPs have been studied: (1) globular polymers with a high fraction of hydrophobic monomers and (2) coil-like polymers enriched in charged monomers. These categories mirror the broad spectrum of protein conformations, from structured globular proteins to intrinsically disordered proteins (IDPs). As such, RHPs can potentially access a range of protein-like functions, spanning from those associated with compact, folded structures to those seen in disordered systems such as biomolecular condensates.

RHPs offer new avenues for the development of functional catalysts. They can be designed as stand-alone catalytic systems or coformulated with natural enzymes to expand operational conditions, protect fragile active sites, or facilitate multistep reaction cascades. Unlike many natural enzymes, RHP-based catalysts can be developed to tolerate harsh environments, such as organic solvents, extreme pH, and elevated temperatures. These polymers can form catalytic microenvironments through intrachain clustering or stimulus-induced conformational changes, enabling reactions under conditions where traditional biocatalysts fail. The incorporation of stimuli-responsive features, such as thermal or pH-responsive functionalities, could also introduce recyclability, making RHP catalysts attractive for industrial applications. Furthermore, coupling RHPs with electro- and photocatalytic systems presents an exciting opportunity to develop hybrid catalytic platforms with improved performance and selectivity.

RHP ensembles also hold promise in biomedical applications, particularly in interfacing with biological systems as functional biomaterials such as hydrogels^80,81^ and polyplex.^82–85^ Their disordered and flexible backbones enable the presentation of multivalent, tunable interaction sites that can modulate protein–protein interactions (PPI), drive or inhibit liquid–liquid phase separation, and stabilize enzymes during manufacturing or storage. These properties of RHPs may offer distinct benefits compared with small molecules in regulating PPI for their greater interaction surfaces.^86^ By tailoring parameters such as charge distribution, hydrophobicity, and sequence randomness, RHPs hold the potential to be developed as excipients for protein and small molecule therapeutics,^87^ carriers for nucleic acid delivery, or stealth coatings to reduce immunogenicity. These principles also underpin the opportunity to design synthetic ion channels, chaperone-like stabilizers, and immunomodulatory agents. When constructed from polypeptide-based backbones, RHPs benefit from their inherent biocompatibility, biodegradability, and structural compatibility with native biological systems, making them particularly well-suited for translational bioapplications.

Finally, the structural versatility of RHPs lends itself to pressing challenges in environmental sustainability and healthcare. Their tunable architectures can be harnessed for the selective enrichment and recovery of rare-earth elements, as well as for capturing viruses and pollutants like per- and polyfluoroalkyl substances (PFAS).^88^ Their synthetic adaptability enables integration into fibers, membranes, beads, or porous scaffolds, making them suitable for on-demand absorption and separation. Moreover, the chemical robustness supports repeated regeneration cycles with minimal performance loss, which is a key requirement for sustainable operations. We envision that RHP-based sorbents and sensors could potentially become powerful tools in sequestration, purification, separation, healthcare, and circular-economy technologies.

### 5.2 Seeking Inspiration from the Dimensionality Reduction of the Protein Universe

While RHPs have shown promising capabilities, a vast landscape of protein functions and specificity remains unexplored, presenting exciting opportunities for future development. Given the complexity of the design space illustrated in Figure 3, navigating this landscape without prior knowledge is difficult. One promising strategy is to draw inspiration from natural protein sequences. If we can instead “compress” the ensemble sequence and structure information on proteins by extracting key features from natural proteins, we may establish simplified synthetic systems that mimic essential protein functions through statistical design principles. Many previous studies have supported this design principle. One of the earliest examples is glatiramer acetate (Copaxone), developed for the treatment of multiple sclerosis. The RHP was designed to mimic the composition of myelin basic protein (MBP), one of the major myelin autoantigens involved in the disease.^89,90^ Similar strategies have also proven successful in the design of cell-permeable, antimicrobial polymers^91^ and adhesive materials.^44^ The key features include but are not limited to tabulated monomer composition and sequence segmental information (e.g., short linear motif or SLiMs) from the Protein Data Bank,^92,93^ or some universal properties such as degree of hydration that is accessible through experimental measurement or computational modeling. This strategy would not only substantially reduce synthesis complexity and cost but also enable high-throughput screening for the rapid discovery of functional materials. Moreover, the fidelity of formulation drift during synthesis, purification, and materialization presents an additional challenge to how well we understand the actual material being applied. We envision that the integration of data-driven discovery, autonomous experimentation, and advanced polymer chemistry will pave the way for this translation. Such a cross-disciplinary research paradigm holds the potential to create entirely new classes of functional materials and offer fresh perspectives on the statistical nature of biomolecular function.

### 5.3 Technical Outlook

While high-throughput and automated^94^ synthesis of polymers is rapidly developing, advancing the development of RHPs requires new synthetic chemistries that expand access to a broader and more diverse design space (Figure 4). Current efforts are largely centered on poly­(acrylate), poly­(methacrylate),^95^ and polypeptide^33,96,97^ platforms that offer useful functionality but cover only a limited portion of the potential chemical landscape. To go beyond these systems, new polymer backbones with varied rigidity, polarity, degradability, or stereochemistry need to be explored.^98^ At the same time, improved control over composition,^58,99^ microstructure, sequence pattern (e.g., gradients and blockiness)^100,101^ is needed to better mimic the local order found in biological systems.^102–104^ Techniques such as flow chemistry and semibatch iterative monomer addition^105–107^ offer promising routes to fine-tune sequence pattern. Coupling these methods with postpolymerization modification can introduce additional functional diversity without compromising scalability. It is essential to prioritize reproducibility during method development. Reliable and consistent polymer synthesis is the foundation for building trustworthy composition-processing-function relationships in RHPs. It is important to rigorously define the synthetic process and verify that the observed functional behaviors are reproducible and not artifacts of batch-to-batch variability.

**Figure 4:**
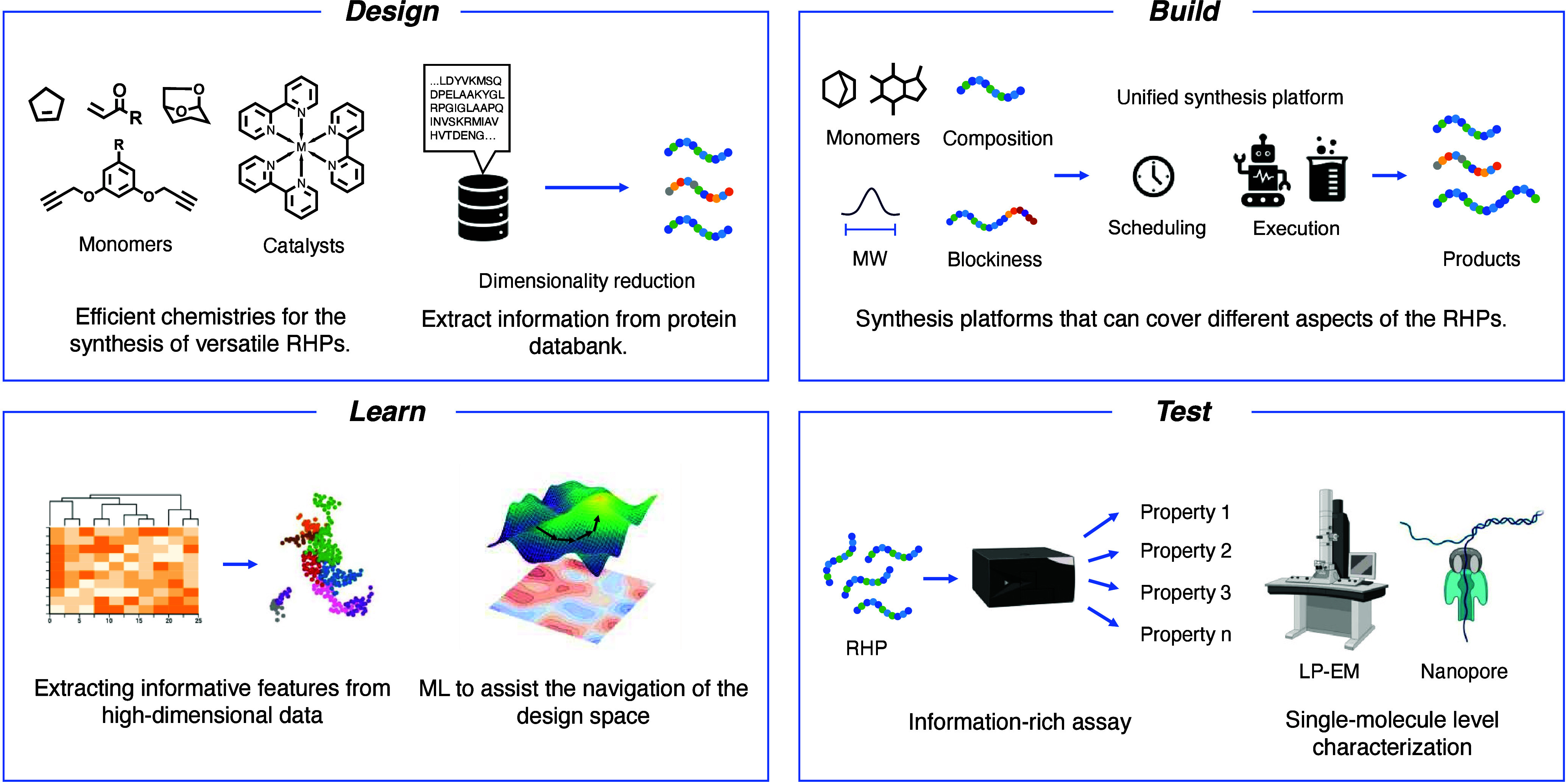
Scheme of the potential developing directions of the design-build-test-learn cycle for RHP materials in the future. In the Design phase, expanding the library of synthetic chemistries will enable efficient access to diverse RHP structures. Additionally, workflows that extract statistical patterns from protein databases, such as amino acid composition and sequence motifs, can help guide the design of functional polymer ensembles. In the Build phase, a unified synthesis platform capable of controlling monomer composition, molecular weight, sequence pattern, and other parameters is essential to systematically exploring the design space. In the Test phase, developing high-throughput, information-rich assays that can simultaneously capture multiple functional properties will support a holistic understanding of RHP behavior. Complementary single-molecule characterization techniques, such as liquid-phase electron microscopy and nanopore analysis, will further elucidate the dynamic behavior and sequence-level features. In the Learn phase, polymer-specific machine learning models are needed to extract meaningful features from high-dimensional experimental data and guide autonomous exploration of the complex, multilevel design space of RHPs.

Moving forward, we hope that the development of ML strategies will facilitate more effective exploration of the hierarchical design space and expedite multi-objective optimization across diverse properties.

Although machine learning (ML) approaches have been successfully applied to guide the design of RHPs by identifying optimal monomer combinations and molecular weights, the design space and initial selection of experiments remain crucial. Instead of relying on a cold start with random initialization, a warm start guided by prior knowledge (Figure 4), such as that from dimensionality reduction of the protein database and computational methods, offers a promising strategy to accelerate the discovery. In addition, high-throughput simulations, ranging from density functional theory to QM/MM and appropriately coarse-grained models that retain necessary physics, can further accelerate and inform the automation pipeline. Moving forward, we hope that the development of ML strategies will facilitate more effective exploration of the hierarchical design space (Figure 3A) and expedite multi-objective optimization across diverse properties.^108^ Meanwhile, physics-informed and interpretable models can extract feature importance and interactions,^109^ enabling retrospective discovery of design rules and structure-function relationships from high-dimensional data.

In parallel, advances in automation are crucial for effectively exploring the RHP design space (Figure 4). High-throughput experimental platforms capable of controlling variables such as monomer composition, polymer chain length, and blend ratios are essential for systematic discovery. These platforms should be paired with intelligent scheduling algorithms^110^ that can dynamically prioritize and sequence experiments based on real-time feedback, enabling efficient navigation of combinatorial spaces. Moreover, while often overlooked, characterization remains a major bottleneck. Developing faster, more informative, and higher-throughput analytical techniques, such as multiplexed assays, in situ spectroscopy, and real-time binding measurements, will significantly enhance the pace of discovery. Ultimately, the integration of automation, advanced ML models, and rapid characterization will be key to accelerating the de novo development of functional RHPs with applications across catalysis, biomedicine, and sustainability.

Coupled with multiscale computational simulations, these experimental approaches can provide comprehensive insights into the ensemble behaviors that drive protein-like functions.

Beyond material discovery, analytical tools are essential for uncovering the underlying mechanisms and advancing the development of RHP materials (Figure 4). Single-molecule-level characterization techniques offer invaluable insights into the structures, dynamics, and heterogeneities of these complex systems. Technologies such as escape-time stereometry (ETS)^111^ and single-molecule FRET^112^ could be potentially useful to quantitatively resolve three-dimensional molecular structures, as well as to extract thermodynamic and kinetic parameters of molecular interactions. Furthermore, nanopore sequencing technologies^113,114^ present exciting possibilities for characterizing the primary structures of the functional polymers within heterogeneous ensembles. Coupled with multiscale computational simulations, these experimental approaches can provide comprehensive insights into the ensemble behaviors that drive protein-like functions.

## Supplementary Material



## Notes

Transparent Peer Review report available (PDF)

## ‡

(G.W.) Current address: Department of Chemistry, University of Oxford, 12 Mansfield Road, Oxford, OX1 3TA, United Kingdom
