# Supplementary material for: Seeking Precise Protein-like Functions from Random Heteropolymer Ensemble and through Dimensionality Reduction
Source: ACS Cent Sci. 2025 Sep 25;11(11):2053–62. doi: 10.1021/acscentsci.5c01382 (PMC12670289; doi:10.1021/acscentsci.5c01382)
Supplement: Supplementary file 1 [file oc5c01382_si_001.pdf]

oc-2025-01382z.R1

Name: Peer Review Information for "Seeking precise protein-like functions from random heteropolymer ensemble and through dimensionality reduction"

First Round of Reviewer Comments

Reviewer: 1

Comments to the Author

This "Outlook" article discusses how random heteropolymers, or RHPs, can serve as synthetic alternatives to proteins by mimicking their diverse biological functions without requiring precise sequence control. It emphasizes recent findings demonstrating that RHPs can catalyze reactions or bind and stabilize proteins. The authors emphasize the importance of data-driven experimentation, machine learning, and multiscale modeling in navigating the vast design space of these polymers and uncovering structure–function relationships. In the future, they suggest that adopting statistical design principles and autonomous workflows could speed up the creation of functional RHPs for use in catalysis, medicine, and sustainability. Overall, the article is well-written and addresses an interesting and original topic. It is of interest to a wide audience interested in chemistry and meets the requirements for publication in ACS Central Science. I also recommend this article for publication, considering the authors' background and the quality of the manuscript. I have the following few comments for the authors.

- In regard to part 2 concerning catalysis, it is of note that macromolecular engineering may also be key to reproducing certain aspects of enzymatic catalysis. This is undoubtedly another significant property of polymers. In this regard, the following reference, Macromol. Biosci. 2024 2400155 would be a useful addition to references 28 and 29 provided by the authors.
- At the molecular level, proteins primarily serve the following biochemical functions: The first is binding and fixation; the second is catalysis; the third is switching capacity; and the fourth is chaperone role. In the second part, the paragraph titled "Other functions"

should more clearly highlight functions 1 and 3 above, particularly by considering the development of numerous switchable polymer systems (stimuli-responsive polymers) or systems capable of multivalent interaction (glycopolymers). I recommend adding a few sentences to this paragraph in this direction.

- The principle of Copaxone-type therapeutic polymers that mimic amino acid distribution has also been developed in the context of cell internalization or membrane destabilization (antimicrobial polymers). It is certainly worth mentioning it in this article.

- Figure 3 is really nice and could be included earlier in the article.

Reviewer: 2

Comments to the Author

This manuscript provides the readers with a short review of recent developments in an emerging area of protein mimics, i.e. random heteropolymers (RHPs) and a good motivation to further advance high throughput synthesis/characterization and leverage capabilities in AI/ML. Although random copolymers have been around for decades, RHPs have received significant attention as protein mimics over the last few years with notable developments by several talented groups in many continents. In many ways, the RHP concept challenges our perception of biology dogma. Their practicality is a bonus. At this stage, this contribution highlights several key areas to move the field forward and will be of interests for a broad range of communities.

Below are a few feedback:

1. The team defines RHPs as heteropolymers with two or more monomers. To delineate from previous studies based on binary monomers (detailed below), many recent studies define RHPs as heteropolymers containing three or more types of monomers. Below is a quite recap of prior work. BTW, these papers should be cited.

Russell and Hawker first experimentally designed and realized random copolymer based on MMA and Styrene. These polymers were named as random copolymers (Science 1997, JACS 1996). Before that (1996-1997), Arup Chakraborty studied recognition of random

copolymers on patterned surfaces. Back in 2001, the late Geissler performed simulation at the single chain level and the system used was called RHPs although there are only two types of monomers.

In Ref. 31, RHPs are based on four monomers. And in ref. 40, RHP was referred to those with three or more monomers.

Since the expanded sequence space with three or more types of monomers and many recent new findings are associated with this sequence space, I personally would suggest reserving RHPs for heteropolymers with three or more types of monomers to help the community to converge on terminology and communication. The binary monomer-based random copolymers should be cited and discussed to set the record straight.

2. A couple of papers from Theresa Reineke were cited from the application point of view. She has done quite a bit more basic studies on RHPs. Although most geared toward her delivery work, I believe the reader will find these studies on RHP alone be useful and should be cited.

3. There have been many studies in biomaterial developments for hydrogel and drug delivery using heteropolymers. Some should be sited. Connecting to these case studies will help readers to connect to specific area(s) of their interests.

Author's Response to Peer Review Comments:

Authors' Response to Reviews of

Seeking precise protein-like functions from random heteropolymer ensemble and through dimensionality reduction

Guangqi Wu, Tianyi Jin, Connor W. Coley, Alfredo Alexander-Katz* and Hua Lu *
*ACS Central Science, *E-mail: aalexand@mit.edu and chemhualu@pku.edu.cn.*

RC: ***Reviewers' Comment***, AR: Authors' Response, ☐ Manuscript Text

Dr. Jeffrey Hubbell
Executive Editor
ACS Central Science

Dear Dr. Jeffrey Hubbell,

We want to first thank you for handling our manuscript (ID: oc-2025-01382z) and also the reviewers for their feedback and positive reception of our work. We have addressed all concerns in this point-by-point response and made the requisite changes to the manuscript. All relevant changes to the manuscript text are reproduced for ease of reference, with new manuscript additions highlighted in blue in this response letter. They are also highlighted in yellow in the resubmitted main text.

We believe that the revised version of our manuscript should merit prompt publication in *ACS Central Science*.

Thank you very much for considering this manuscript and we are looking forward very much to hearing from you soon.

Best wishes,

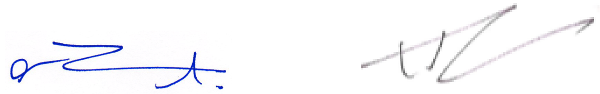

Alfredo Alexander-Katz and Hua Lu (on behalf of all authors)

Reviewer 1

RC: ***This "Outlook" article discusses how random heteropolymers, or RHPs, can serve as synthetic alternatives to proteins by mimicking their diverse biological functions without requiring precise sequence control. It emphasizes recent findings demonstrating that RHPs can catalyze reactions or bind and stabilize proteins. The authors emphasize the importance of data-driven experimentation, machine learning, and multiscale modeling in navigating the vast design space of these polymers and uncovering structure–function relationships. In the future, they suggest that adopting statistical design principles and autonomous workflows could speed up the creation of functional RHPs for use in catalysis, medicine, and sustainability. Overall, the article is well-written and addresses an interesting and original topic. It is of interest to a wide***

audience interested in chemistry and meets the requirements for publication in ACS Central Science. I also recommend this article for publication, considering the authors' background and the quality of the manuscript. I have the following few comments for the authors.

AR: We thank the reviewer for their thoughtful feedback and have revised the manuscript accordingly to incorporate the suggested comments.

RC: ***In regard to part 2 concerning catalysis, it is of note that macromolecular engineering may also be key to reproducing certain aspects of enzymatic catalysis. This is undoubtedly another significant property of polymers. In this regard, the following reference, Macromol. Biosci. 2024 2400155 would be a useful addition to references 28 and 29 provided by the authors.***

AR: We have added this reference to the manuscript along with a brief description.

> Catalytic functionalities can be introduced either through reactive monomers or the initiators themselves during the polymerization\cite {terashima2011single}\cite {tronnet2024star} or via post-polymerization modification \cite {stals2013balance}. The catalytic activities could be tuned by various parameters such as the monomer compositions and the architecture of the polymers. RHPs have also demonstrated catalytic activity across a range of reactions.

RC: ***At the molecular level, proteins primarily serve the following biochemical functions: The first is binding and fixation; the second is catalysis; the third is switching capacity; and the fourth is chaperone role. In the second part, the paragraph titled "Other functions" should more clearly highlight functions 1 and 3 above, particularly by considering the development of numerous switchable polymer systems (stimuli-responsive polymers) or systems capable of multivalent interaction (glycopolymers). I recommend adding a few sentences to this paragraph in this direction.***

AR: We have added the relevant references on stimuli-responsive polymers and glycopolymers to the manuscript.

> The Gong group recently developed high-performance underwater adhesive hydrogels that mimic the function of natural adhesive proteins by integrating data mining from protein databases with experimentation and machine learning \cite {liao2025data}. These materials highlight the potential of RHP-inspired systems to achieve strong and selective binding through multivalent interactions, paralleling one of the core biochemical functions of proteins. In addition, stimuli-responsive behaviors, such as temperature sensitivity \cite {shibata2021thermoresponsive,chittari2024structure}, have been introduced into RHP systems, enabling polymers to act as molecular switches. Meanwhile, introducing glycans into RHPs has emerged as a powerful strategy to mimic glycoproteins, providing a versatile platform for studying glycan–protein interactions and designing functional biomaterials for applications in therapeutic delivery, infection models, and vaccine development\cite {kohout2022synthesis}.

RC: ***The principle of Copaxone-type therapeutic polymers that mimic amino acid distribution has also been developed in the context of cell internalization or membrane destabilization (antimicrobial polymers). It is certainly worth mentioning it in this article.***

AR: We have added the relevant references to the manuscript.

> Many previous studies have supported this design principle. One of the earliest examples is glatiramer acetate (Copaxone®), developed for the treatment of multiple sclerosis. This random copolymer was designed to mimic the composition of myelin basic protein (MBP), one of the major

myelin autoantigens involved in the disease \cite {arnon1996development,arnon2004mechanism}. Similar strategies have also proven successful in the design of cell-permeable, antimicrobial polymers \cite {takahashi2017synthetic} and adhesive materials \cite {liao2025data}.

RC: ***Figure 3 is really nice and could be included earlier in the article.***

AR: We have moved Figure 3 to the beginning of section 3 (outlook).

Reviewer 2

RC: ***This manuscript provides the readers with a short review of recent developments in an emerging area of protein mimics, i.e. random heteropolymers (RHPs) and a good motivation to further advance high throughput synthesis/characterization and leverage capabilities in AI/ML. Although random copolymers have been around for decades, RHPs have received significant attention as protein mimics over the last few years with notable developments by several talented groups in many continents. In many ways, the RHP concept challenges our perception of biology dogma. Their practicality is a bonus. At this stage, this contribution highlights several key areas to move the field forward and will be of interests for a broad range of communities.***

AR: We thank the reviewer for their thoughtful feedback and have revised the manuscript accordingly to incorporate the suggested comments.

RC: ***The team defines RHPs as heteropolymers with two or more monomers. To delineate from previous studies based on binary monomers (detailed below), many recent studies define RHPs as heteropolymers containing three or more types of monomers. In Ref. 31, RHPs are based on four monomers. And in ref. 40, RHP was referred to those with three or more monomers. Since the expanded sequence space with three or more types of monomers and many recent new findings are associated with this sequence space, I personally would suggest reserving RHPs for heteropolymers with three or more types of monomers to help the community to converge on terminology and communication. The binary monomer-based random copolymers should be cited and discussed to set the record straight.***

AR: We have adapted the following revisions to clarify this point.

> More recently, random heteropolymers (RHPs), synthesized through the statistical copolymerization of three or more monomer types, have recently regained attention as promising candidates for mimicking and interacting with proteins.

> These seminal theoretical and experimental studies were primarily based on binary monomer systems, whereas more recent advances have extended to heteropolymers with three or more monomers, thereby broadening the accessible sequence space and functional scope. This enormous chemical design space holds significant potential for discovering new functions and materials through variations in monomer and composition.

RC: ***Below is a quite recap of prior work. BTW, these papers should be cited. Russell and Hawker first experimentally designed and realized random copolymer based on MMA and Styrene. These polymers were named as random copolymers (Science 1997, JACS 1996). Before that (1996-1997), Arup Chakraborty***

studied recognition of random copolymers on patterned surfaces. Back in 2001, the late Geissler performed simulation at the single chain level and the system used was called RHPs although there are only two types of monomers.

AR: We have added the relevant references to the manuscript.

> This has positioned them at a fascinating intersection between polymer science \cite {mansky1997controlling,mansky1997interfacial} and protein science \cite {pande2000heteropolymer,chakraborty2001disordered,geissler2002reversible}, making it a hot topic since the 1990s and one that will continue to attract significant interest in the future.

RC: ***A couple of papers from Theresa Reineke were cited from the application point of view. She has done quite a bit more basic studies on RHPs. Although most geared toward her delivery work, I believe the reader will find these studies on RHP alone be useful and should be cited.***

AR: We have added Reineke's papers on fundamental studies, as well.

> Unlike block copolymers \cite {sprouse2014investigating}, RHPs are inherently complex systems; each polymer chain differs from others in terms of sequence, composition,...

> At the same time, improved control over composition \cite {ting2013precise,yu2024mapping}, microstructure,

RC: ***There have been many studies in biomaterial developments for hydrogel and drug delivery using heteropolymers. Some should be sited. Connecting to these case studies will help readers to connect to specific area(s) of their interests.***

AR: We have added the relevant references on biomaterials.

> The Gong group recently developed high-performance underwater adhesive hydrogels that mimic the function of natural adhesive proteins by integrating data mining from protein databases with experimentation and machine learning \cite {liao2025data}.

> RHP ensembles also hold promise in biomedical applications, particularly in interfacing with biological systems as functional biomaterials such as hydrogels \cite {podual2000preparation,dai2006temperature} and polyplex \cite {kumar2020efficient,gurnani2020vitro,rui2022high,patel2023core}.

> By tailoring parameters such as charge distribution, hydrophobicity, and sequence randomness, RHPs hold the potential to be developed as excipients for protein and small molecule therapeutics \cite {ting2018advances}, carriers for nucleic acid delivery, or stealth coatings to reduce immunogenicity.

Additional revisions

AR: We have revised our paper (Wu et al., 2025) and included an additional study to highlight the importance of considering scheduling when optimizing pipetting time (Wu et al., 2025).

We recently developed a self-driving experimental platform coupled with a model-free genetic algorithm (GA) \cite {wu2025autonomous}.

These platforms should be paired with intelligent scheduling algorithms\cite {wu2025optimization} that can dynamically prioritize and sequence experiments based on real-time feedback, enabling efficient navigation of combinatorial spaces.

AR: We have included an additional reference to demonstrate the importance of synthetic accessibility, specifically whether certain sequences can be realized through particular polymerization techniques.

Moving forward, we hope that the development of ML strategies can explore the hierarchical design space (Figure 3A) more effectively and conduct multi-objective optimization across diverse properties simultaneously, while remaining synthetically accessible through different polymerization techniques\cite {guzman2025chainspace}.

AR: We have included the following Pull Quotes.

1. Instead of emulating a single, precisely folded macromolecule, RHPs can be engineered as ensembles whose collective behavior yields protein-like functions

3. Coupled with multiscale computational simulations, these experimental approaches can provide comprehensive insights into the ensemble behaviors that drive protein-mimicking functions.

3. Compared to rational design, data-driven autonomous experimentation is particularly powerful to distill unexpected formulation-function relationships in complex systems where little prior knowledge exists

4. Moving forward, we hope that the development of ML strategies can explore the hierarchical design space more effectively and conduct multi-objective optimization across diverse properties simultaneously.
